# Sleep Stage Classification Using Time-Frequency Spectra From Consecutive Multi-Time Points

**DOI:** 10.3389/fnins.2020.00014

**Published:** 2020-01-28

**Authors:** Ziliang Xu, Xuejuan Yang, Jinbo Sun, Peng Liu, Wei Qin

**Affiliations:** Engineering Research Center of Molecular and Neuro Imaging of Ministry of Education, School of Life Sciences and Technology, Xidian University, Xi’an, China

**Keywords:** sleep stage classification, deep learning, electroencephalogram, long short-term memory network, time-frequency spectrum

## Abstract

Sleep stage classification is an open challenge in the field of sleep research. Considering the relatively small size of datasets used by previous studies, in this paper we used the Sleep Heart Health Study dataset from the National Sleep Research Resource database. A long short-term memory (LSTM) network using a time-frequency spectra of several consecutive 30 s time points as an input was used to perform the sleep stage classification. Four classical convolutional neural networks (CNNs) using a time-frequency spectra of a single 30 s time point as an input were used for comparison. Results showed that, when considering the temporal information within the time-frequency spectrum of a single 30 s time point, the LSTM network had a better classification performance than the CNNs. Moreover, when additional temporal information was taken into consideration, the classification performance of the LSTM network gradually increased. It reached its peak when temporal information from three consecutive 30 s time points was considered, with a classification accuracy of 87.4% and a Cohen’s Kappa coefficient of 0.8216. Compared with CNNs, our results indicate that for sleep stage classification, the temporal information within the data or the features extracted from the data should be considered. LSTM networks take this temporal information into account, and thus, may be more suitable for sleep stage classification.

## Introduction

Sleep is an indispensable part of our life and occupies about one third of our total lifetime (Cirelli and Tononi, 2008). Sleep not only reduces tiredness from daily life, but during sleep, our brain and other organs are also repaired (Oswald, 1980). However, with the pace of life becoming faster and faster, the stresses that people experience are gradually increasing, and sleep time and quality are gradually decreasing (Geiker et al., 2018). These factors cause a series of sleep-related diseases, including hypertension (Palagini et al., 2013), angiocardiopathy (Cowie, 2017), and depression (Roberts and Duong, 2014). Thus, better sleep is necessary for a normal life.

Generally, besides asking people about their sleep, doctors also judge sleep quality by acquiring and analyzing electroencephalography (EEG) data from an entire night. As this method requires calculating the ratio between the time of each sleep stage and the total sleep duration, a crucial aspect of this method is the precision of sleep stage classification. However, for EEG data from a typical night, the average total duration is about 6–8 h, meaning a relatively large workload for a technician to manually classify the data. Generally, an experienced technician can only complete the EEG datasets for 3–4 entire nights in 1 day. Considering the tiredness of the technician and other subjective factors, manual classification is not an efficient approach and easily results in mistakes being made. Thus, an automatic sleep stage classification method is needed.

Deep learning is one popular kind of artificial intelligence algorithm. It has been widely applied in many fields and has achieved many remarkable results (Cai et al., 2016; Kim et al., 2017; Liu et al., 2017, 2018; Sharma et al., 2017; Zhang and Zhou, 2018). Recently, studies of deep learning-based automatic sleep stage classification have also frequently been published (Fiorillo et al., 2019). Zhang and Wu (2018) proposed a complex value convolutional neural network (CCNN) based an automatic sleep stage classification method. In this method, a single channel of EEG data was firstly converted into a complex value format. Then, a CCNN was constructed to perform the classification work with the complex EEG data as the input (Zhang and Wu, 2018). Zhang and Wu (2017) subsequently improved the CCNN and proposed the Fast Discriminate CCNN. Bresch et al. (2018) and Supratak et al. (2017) both proposed a sleep stage classification method which combines a convolutional neural network (CNN) and a long short-term memory (LSTM) network. Bresch et al. (2018) used a CNN to extract features every 30 s from a single-channel EEG signal and used an LSTM network to perform the classification with the extracted features as the input. Supratak et al. (2017) directly calculated features from the EEG data. After selecting features using the CNN, the remaining features were used as inputs for the LSTM network. Michielli et al. (2019) proposed a cascaded LSTM networks-based sleep classification method. This method first combines rapid eye moment (REM) sleep and non-REM stage 1 (N1) sleep into one class, and then uses an LSTM 1 network to classify the additional four sleep stages. Next, the N1–REM sleep class selected by the LSTM 1 network is used as the input into the LSTM 2 network and is further classified as N1 sleep or REM sleep. The sleep stage classification performance values reported by these studies were all good; however, there are still two problems: (1) the relatively small size of the datasets used; and (2) the fact that the temporal information shared between 30 s EEG data time points was not considered.

Chambon et al. (2018) proposed a CNN-based sleep stage classification method that considers temporal relationships in the EEG data. This method first extracts features from each 30 s epoch of the EEG data using a CNN. Next, features from three consecutive 30 s EEG data epochs (the 30 s before, the current 30 s, and the 30 s after) are combined to make the final classification decision. Inspired by this study, in this paper, we use an LSTM model to perform sleep stage classification using the time-frequency spectra from several continuous 30 s EEG data epochs as the input. To address the problem of dataset size, we used the Sleep Heart Health Study dataset from the National Sleep Research Resource database (Quan et al., 1997; Redline et al., 1998; Dean et al., 2016; Zhang et al., 2018).

## Materials and Methods

### Dataset

We used the Sleep Heart Health Study (SHHS) dataset from the National Sleep Research Resource database^1^. This dataset contains two sub-datasets, named SHHS1 and SHHS2. SHHS1 contains one night of sleep EEG data for 5,793 subjects, collected between November 1st, 1995 and January 31st, 1998 (Visit 1). SHHS2 contains one night of sleep EEG data for 2,651 subjects, collected between January 2001 and June 2003 (Visit 2); these are the second acquisition time points for a subset of the SHHS1 subjects. All the EEG datasets contain C3, C4, electrooculography (EOG) L, EOG R, and electromyography (EMG) channels, and nine other heart rate related channels. Considering the large number of subjects in each of these two datasets, we used the SHHS1 dataset for training and the SHHS2 dataset for testing.

### EEG Data Preprocessing

**FIGURE 1:**
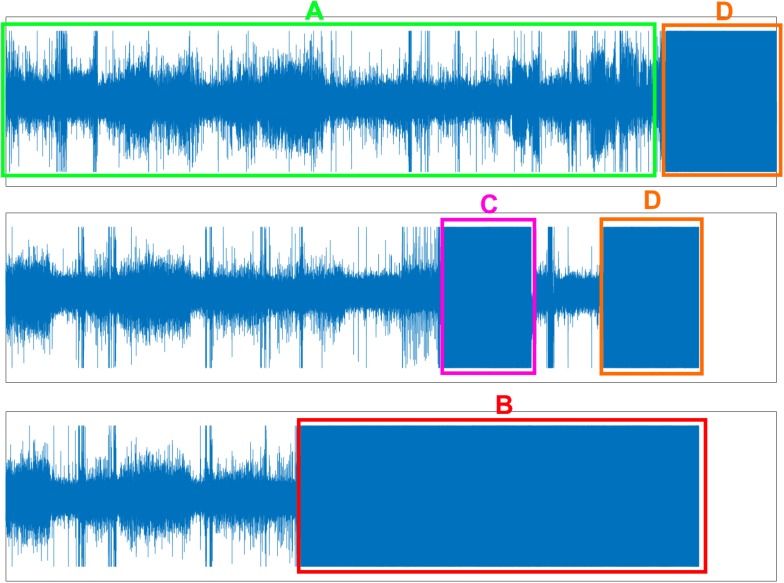
Three problems of EEG data. **(A)** Normal EEG data (green box). **(B)** Electrode dropped (red box). **(C)** Electrode dropped temporally (pink box). **(D)** Removing electrode in the morning but forgetting turn-off the acquisition device (orange box).

All the EEG data in these two datasets were first checked for problems with electrode dropping (Figure 1B). Briefly, the average absolute amplitude of the C3, C4, EOG L, EOG R, and EMG channels across the entire night were calculated. If the average amplitude of a channel was larger than a predetermined threshold, the electrode of this channel was identified as “dropped” and was excluded. In this study, we set the threshold of each channel to half of the maximum physical acquisition amplitude of each channel, which was obtained from the header of each EEG data file.

Secondly, band-pass filtering was performed on each non-dropped channel. For the C3 and C4, EOG L and EOG R, and EMG channels, the filtering frequency ranges were set to 0.3–45 Hz, 0.3–12 Hz, and 0.3–20 Hz, respectively (Patanaik et al., 2018). We used a 50th order Hamming window-based finite impulse response (FIR) band-pass filter to zero-phase filter the data. Briefly, the EEG data were firstly filtered by the FIR filter. Next, the output was reversed and was filtered again by the FIR filter. Finally, the output was reversed again to yield the zero-phase filtering results. The zero-phase filtering preserves the phase information of EEG data while performing filtering on the data.

Finally, the filtered data was checked for problems with temporary electrode dropping and for continued EEG data acquisition after the electrodes were removed from the participant in the morning (Figures 1C,D). Similar to the protocol used to check for the electrode dropping problem, in each 30 s data epoch, the average absolute amplitudes of the C3, C4, EOG L, EOG R, and EMG channels were calculated. If the average amplitudes of both the C3 and C4 channels, or both the EOG L and EOG R channels, or the EMG channel were larger than the maximum physical acquisition amplitude of each channel, the corresponding 30 s data epoch was excluded. Figure 1A displayed the normal EEG data which were used for further analysis.

### Feature Extraction

In this study, the time-frequency spectra for each 30 s data epoch was used as the input for sleep stage classification (Patanaik et al., 2018). Time-frequency spectra for each 30 s data epoch were calculated from four of the channels (EEG, EOG L, EOG R, and EMG).

For the EEG channel, the averaged data of the C3 and C4 channels were used to compute the spectra. If one of these two channels had a problem with electrode dropping, the data of the non-dropped channel was used. For the two EOG channels, if one channel was dropped, then both EOG L and EOG R channel used the data from the non-dropped channel to compute the spectra. These two protocols were used to maximize the number of training and testing samples and to make the classification model better adapted to the situation that occurs during real sleep.

**FIGURE 2:**
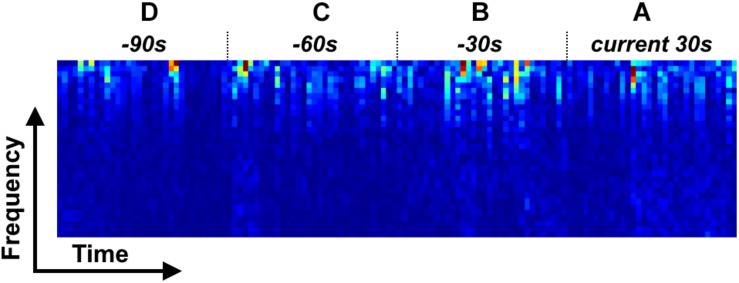
Four types of time-frequency spectrum stack for LSTM model. **(A)** LSTM 1; **(A,B)** LSTM 2; **(A–C)** LSTM 3; **(A–D)** LSTM 4.

To compute the spectra for each channel, each 30 s data epoch was first resampled to 100 Hz, resulting in 3,000 data points for each 30 s data epoch. Secondly, a short-time Fourier transformation (STFT) was performed on each 30 s epoch of the resampled data. A Hamming window of length 128 with an overlap of 38 points (i.e., with a slide step of 90 points) was used and the fast Fourier transformation (FFT) was applied to yield the time-frequency spectra. This resulted in a time-frequency spectra with 32 time points (with a resolution of 900 ms) by 65 frequency points (with a resolution of 0.7752 Hz). Only the first 32 frequency bins (0–24 Hz) were analyzed, which resulted in a 32 × 32 time-frequency spectra for each 30 s resampled data epoch. Next, for each time point in the spectra, the frequency points were normalized to the range 0–1 (Figure 2). Finally, the time-frequency spectra of the EEG, EOG L, EOG R, and EMG channels were stacked to yield a four-channel matrix of size 32 × 32 × 4 and this matrix was used as the input feature of a classification model.

### Classification Model

**FIGURE 3:**
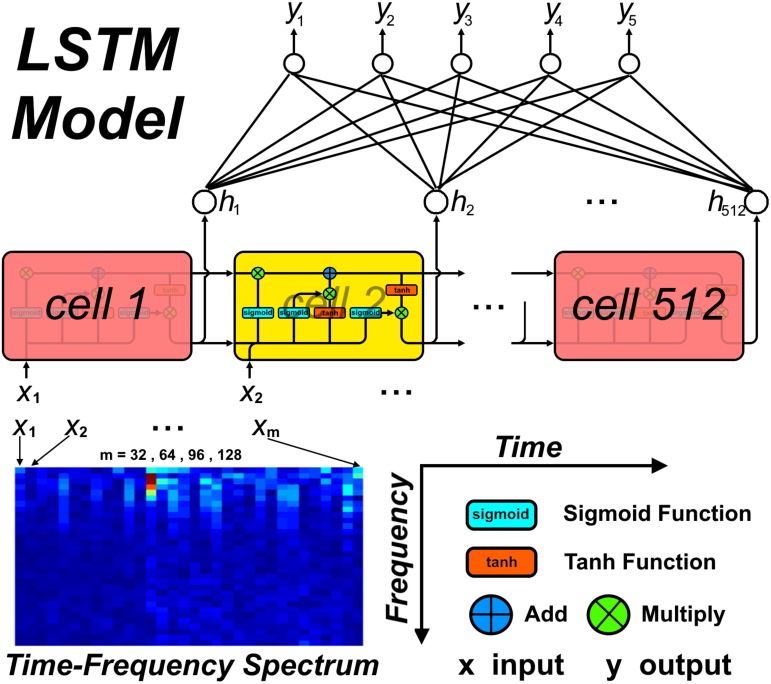
The workflow of study procedure.

In this study, a LSTM network (Hochreiter and Schmidhuber, 1997) was used to build a classification model, with a spectral stack of four channels as the input. We considered four types of input mode to investigate the performance of the LSTM network at classifying sleep stages: (1) a single 30 s spectral stack, for which the network only considers the temporal information within each current 30 s epoch (Figure 2A); (2) two consecutive 30 s spectral stacks, for which the network considers the temporal information in the previous 30 s epoch and the current 30 s epoch (Figures 2A,B); (3) three consecutive 30 s spectral stacks, for which the network considers the temporal information in the two previous 30 s epochs and the current 30 s epoch (Figures 2A–C); and (4) four consecutive 30 s spectral stacks, for which the network considers the temporal information in the three previous 30 s epochs and the current 30 s epoch (Figures 2A–D). For convenience, the LSTM networks with input modes 1–4 are hereafter termed the LSTM 1, LSTM 2, LSTM 3, and LSTM 4 networks, respectively. Because the input mode we used only considers the time information from previous epochs and the current epoch, our constructed network can be used for both online and offline sleep stage classification.

For comparison, the CNN from Patanaik et al. (2018) (Normal CNN), and the well-known CNNs AlexNet (Krizhevsky et al., 2012), VGG16 (Simonyan and Zisserman, 2015) and GoogLeNet (Szegedy et al., 2015) were used for classification model building. The input of each of these models was a single 30 s spectral stack. The detailed network structure of each of these four CNNs can be found in Supplementary Tables S1–S3.

### Experimental Setup

The problem of sleep stage classification was approached as a five-class classification problem, with the following classes: 0 for the waking state; 1 for the N1 sleep stage; 2 for the N2 sleep stage; 3 for the N3 and N4 sleep stages; and 4 for the REM sleep. For each input spectral stack from one 30 s EEG data epoch, if the classification model identified this input as being of class 0–4, then the sleep stage of this 30 s EEG data epoch would be set to 0–4 (i.e., the sleep stage was detected every 30 s).

For the LSTM network, the number of cells was set to 512, which would result in 512 outputs at the end of the network. Thus, a fully connected layer was added to the end of the LSTM network, which transformed the number of outputs to 5. For each input mode, the spectra from the four EEG channels were combined along the frequency axis, i.e., the (*i* × 32) × 32 × 4 spectral stack was reshaped to a (*i* × 32) × 128 spectrum, with *i* = 1–4 corresponding to the four input modes. For the CNNs, the 32 × 32 × 4 spectral stack was input directly into the networks. Figure 3 shows the detailed workflow of the LSTM network.

For each input mode, a mini-batch was first constructed. The size of the mini-batch was set to 4,096, according to the GPU memory size used. Considering the memory size of our workstation, we used the random splitting method to create each 4,096 mini-batch. Briefly, each subject index in the training and testing dataset was randomly permuted, independently of each other. After that, the permuted training and testing datasets were split into *N*_train_ and *N*_test_ groups, with each group containing *Num*_train_ and *Num*_test_ numbers of subjects. Next, the entire time-frequency spectral stacks from each subject in each splitting group were randomly permuted, independently in each splitting group, and used to create several 4,096 mini-batches. If the number of time-frequency spectral stacks in one group could not be exactly divided by 4,096, the size of the last mini-batch in this group was set to the remainder. Finally, all of these smaller mini-batches were combined to create several 4,096 mini-batches, but this time, the remainder mini-batch was excluded. In total, 1,324 training mini-batches and 684 testing mini-batches were constructed, containing about 5,324,000 training samples and 2,457,600 testing samples.

A ReLU function was used as the activation function for the CNNs. Considering the unbalanced numbers of samples in each of the five sleep classes, a weighted softmax cross entropy logit function was used as the cost function for each network (Xie and Tu, 2017). An Adam optimization algorithm (Kingma and Ba, 2015) with 0.9 and 0.999 exponential decay rates for the 1st and the 2nd moments, respectively, was used for training each network, and the maximum number of iterations was set to 200 epochs. The initial learning rate was set to 0.001 and was divided by 10 every 50 epochs. Training was stopped under the following conditions: (1) if the number of epochs reached the maximum; (2) if an absolute cost difference of less than 1e-5 occurred between two successive testing epochs on five successive occasions; or (3) if the testing cost increased across five successive epochs.

To investigate the effect of mini-batch size on the classification results, for each network (CNN and LSTM), a series of mini-batch sizes were used (4,096, 2,048, 1,024, 512, 256, and 128). For each model, the mini-batch size was set to the specific value that yielded the maximum accuracy index. As the mini-batch size of each batch created using the method described above was 4,096, the other mini-batch sizes were created by splitting the 4,096 mini-batch into several component mini-batches. For example, a 4,096 mini-batch was split into two 2,048 mini-batches, or four 1,024 mini-batches, and so on. Detailed hyper-parameter settings for each network can be found in Table 1.

**TABLE 1:** The hyper-parameters setting for each network.

**Stage**	**Name**	**Normal CNN**	**AlexNet**	**VGG16**	**GoogLeNet**	**LSTM**
initialization	Bias	0				
	Weight	Gaussian				
	Active function	ReLU	ReLU	ReLU	ReLU	–
	cost function	Softmax with logit entropy				

Training	Maximum epochs	200				
	initial learning rate	1e-3				
	final learning rate	1e-6				
	Mini-batch size	2048	1024	2048	1024	1024
	Dropout rate	0.5	0.5	0.5	0.5	–
	Optimization	Adam				

Training and testing of the CNN and LSTM models were implemented using TensorFlow 1.12 with GPU support on a Python 3.5 platform.

### Evaluation Index

In this study, the accuracy index, sensitivity index and Cohen’s Kappa (CK) coefficient were used to evaluate our proposed method. The accuracy index was used to evaluate the overall sleep stage classification accuracy of the network, while the sensitivity index focused on the classification accuracy of each sleep stage. The CK coefficient was used to evaluate the overall sleep stage classification performance of the network. Detailed definitions of these three indices are as follows:

(1) $$\text{Accuracy}=\frac{\mathrm{number}\,\mathrm{of}\,\mathrm{accurately}\,\mathrm{scored}\,\mathrm{epochs}}{\mathrm{total}\,\mathrm{number}\,\mathrm{of}\,\mathrm{epochs}}$$

(2) $${\text{Sensitivity}}_{i}=\frac{\mathrm{number}\,\mathrm{of}\,\mathrm{accurately}\,\mathrm{scored}\,\mathrm{epochs}\,i\,n\,\mathrm{stage}\,i\,}{\mathrm{number}\,\mathrm{of}\,\mathrm{epochs}\,\mathrm{in}\,\mathrm{stage}\,i}$$

(3) $$\text{Kappa}=\frac{\text{Accuracy}-P_{e}}{1-P_{e}}$$

(4) $$\begin{array}{c} P_{e}=\frac{\sum_{i}a_{i}\cdot b_{i}}{n\,u\,m^{2}}\\ i\in \left[\mathrm{wake},\mathrm{N1},\mathrm{N2},\mathrm{N3},\mathrm{REM}\right] \end{array}$$

Where, *a*_*i*_ represents the real number of epochs in stage *i*; *b*_*i*_ represents the number of epochs predicted in stage *i* by the networks; and *num* represents the total number of epochs.

## Results

### CNN Performance

**FIGURE 4:**
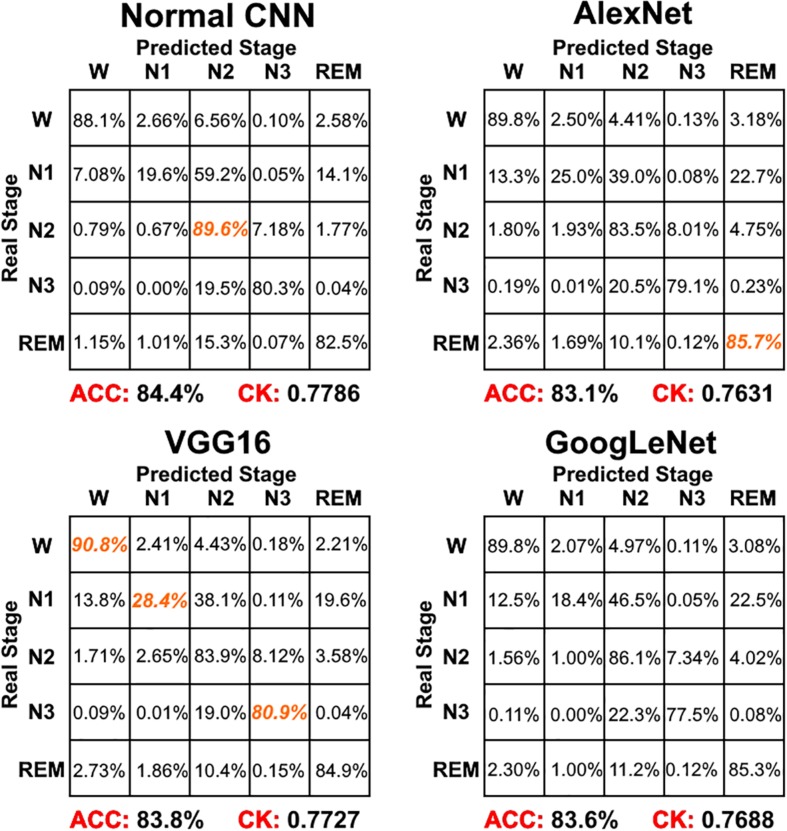
The sleep stage classification performance for each CNN network. Each row represents the percent of a sleep stage that was accurately predicted. The diagonal elements represent the sensitivity index of the network for each sleep stage. ACC represents the accuracy index of the network. CK represents the Cohen’s Kappa coefficient of the network. Orange color represents the maximum sensitivity index of each sleep stage among four networks in figure.

**FIGURE 5:**
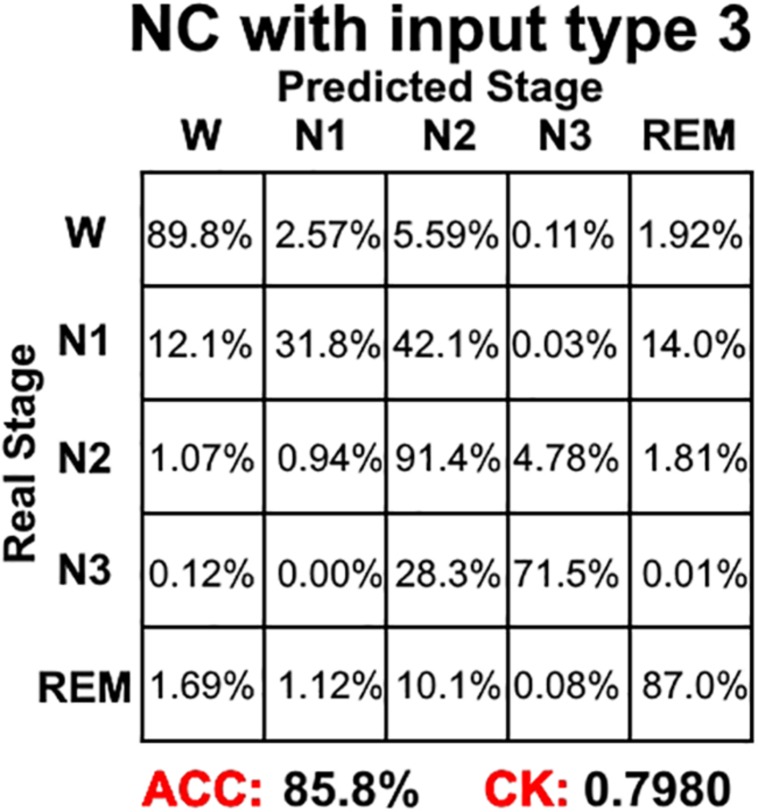
The sleep stage classification performance for Normal CNN with input mode 3. Each row represents the percent of a sleep stage that was accurately predicted. The diagonal elements represent the sensitivity index of the network for each sleep stage. ACC represents the accuracy index of the network. CK represents the Cohen’s Kappa coefficient of the network.

Figure 4 shows the sleep stage classification performance for each CNN. From the figure, it can be seen that the Normal CNN had the simplest structure, but the best classification performance, with an accuracy of 84.4% and a CK coefficient of 0.7786. Figure 5 shows the sleep stage classification performance for the Normal CNN with input mode 3. From the figure, it can be seen that the accuracy and CK coefficient of Normal CNN with input mode 3 were increased to 85.8% and 0.7980, respectively, when more features were input into the network.

### LSTM Network Performance

**FIGURE 6:**
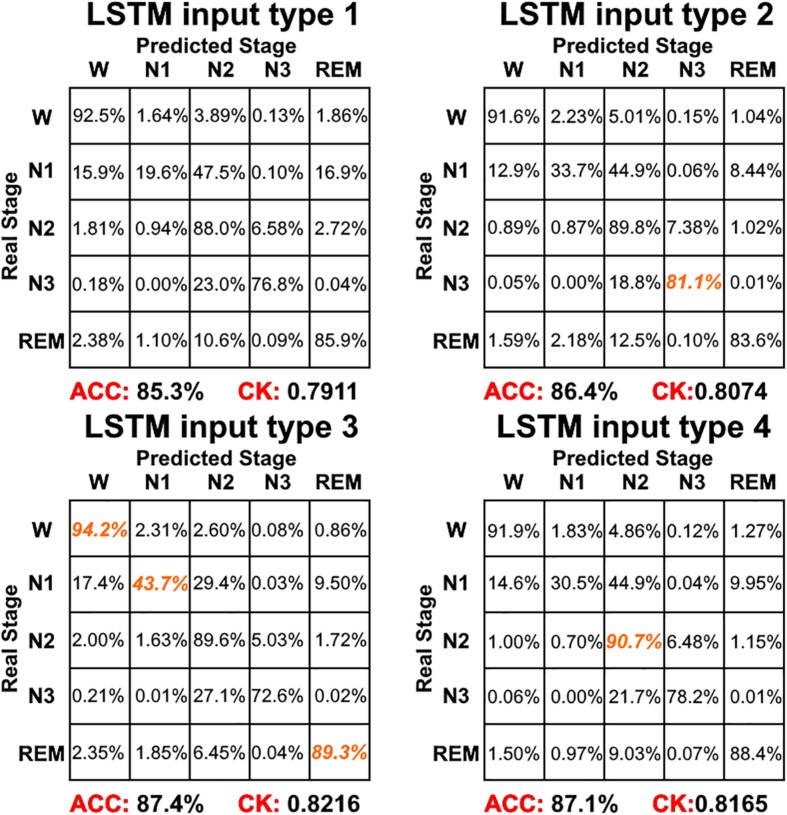
The sleep stage classification performance for each LSTM network. Each row represents the percent of a sleep stage that was accurately predicted. The diagonal elements represent the sensitivity index of the network for each sleep stage. ACC represents the accuracy index of the network. CK represents the Cohen’s Kappa coefficient of the network. Orange color represents the maximum sensitivity index of each sleep stage among four networks in figure.

**FIGURE 7:**
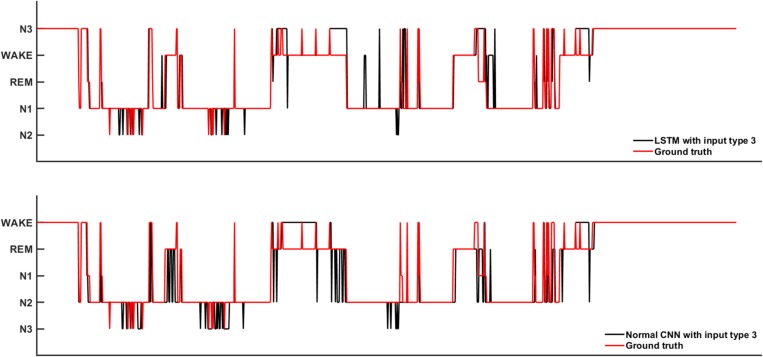
The real sleep stage classification results from one subjects. Ground truth means the real sleep stage label.

Figure 6 shows the sleep stage classification performance for the LSTM network. Compared with Figure 4, the performance of the LSTM 1 network was better than the Normal CNN network on all stages, except for the N2 and N3 stages. Furthermore, the LSTM 1 network had a better classification accuracy and a better CK coefficient than any of the CNNs, with values of 85.3% and 0.7911, respectively. Compared with Figure 5, the classification performance of LSTM 1 also approximated to the performance of Normal CNN with input mode 3 (CK coefficient was only less 0.0069 than that of Normal CNN with input mode 3), which further implied the potential advantage of time information. Moreover, when two consecutive 30 s epochs (1 min) of temporal information were considered at a time from the time-frequency spectral stack, the sensitivity index of the LSTM 2 network was almost better than Normal CNN with input mode 3, except for the N2 and REM stage, and the classification accuracy and the CK coefficient also increased to 86.4% and 0.8074, respectively. When three consecutive 30 s epochs (1 min 30 s) of temporal information were considered at a time from the time-frequency spectral stack, the performance of the LSTM 3 network again increased a little bit. However, when four consecutive 30 s epochs (2 min) of temporal information were considered at a time from the time-frequency spectral stack, the performance of the LSTM 4 network was lower. For the waking, N1 and REM stages, LSTM 3 had the best sensitivity with values of 94.2, 43.7, and 89.3%, respectively. For the N2 stage, LSTM 3 had a sensitivity of 89.6%, which was only 0.9% less than the best-performing network among LSTM networks. Although for the N3 stage, LSTM 3 had the worst sensitivity with a value of 72.6%, its classification accuracy and CK coefficient were the highest at 87.4% and 0.8216, respectively. Thus, after this comprehensive consideration, these results suggest that the LSTM 3 network has the best performance among all the networks mentioned above. Figure 7 shows real classification results from an entire night of EEG data from one subject using the Normal CNN with input mode 3 and LSTM 3 networks.

**FIGURE 8:**
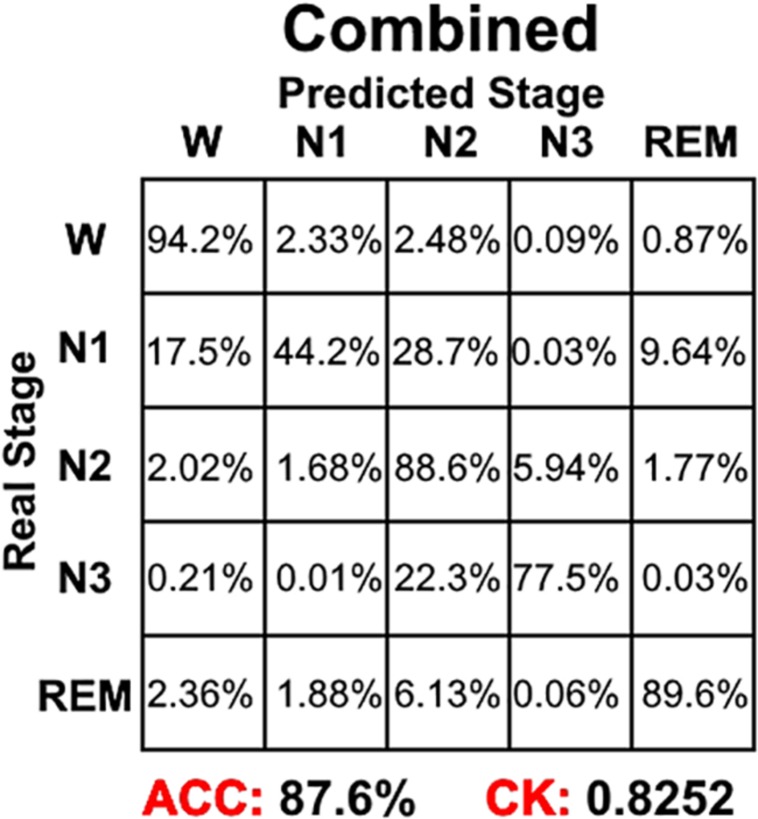
The sleep stage classification performance for combined LSTM network. Each row represents the percent of a sleep stage that was accurately predicted. The diagonal elements represent the sensitivity index of the network for each sleep stage. ACC represents the accuracy index of the network. CK represents the Cohen’s Kappa coefficient of the network.

Combining the information from Figures 4–6, it can be seen that LSTM 3 had the best classification accuracy and CK coefficient of any of the networks mentioned above, but it also had the worst sensitivity for the N3 stage. From all the networks, LSTM 2 had the best sensitivity for the N3 stage. Thus, similar to processing step in the study by Chambon et al. (2018), we combined the predicted probability of each sleep stage from the LSTM 2 and LSTM 3 networks to make the final decision. The detailed decision criteria were as follows: (1) samples which were identified as N2 or N3 by the LSTM 3 network were input into the LSTM 2 network and (2) if the identified maximum probability of LSTM 3 was larger than that of LSTM 2, the final decision would be the one identified by LSTM 3, and vice versa. The results showed that, compared with LSTM 3, although the sensitivity of the combined network on the N2 stage decreased a little bit, the sensitivity on the other stages increased, especially for the N3 stage, and the classification accuracy and CK coefficient also increased by 0.2% and 0.0036, respectively (Figure 8). Thus, this method of combining networks would be one direction to consider in a future study.

### Calculation Efficiency

Using a NVIDIA GeForce GTX Titan X Pascal GPU on an Intel Core i7-6900K PC, it took about 30–40 hours to train a CNN or LSTM network due to the complexity of each model. In the testing stage, a 4,096 mini-batch took 2 s to test; thus, sleep stage classification for an entire night of data can be performed for 2 subjects every second.

## Discussion

In this study, we proposed an LSTM model for sleep stage classification with different durations of time-frequency spectral data as the input. For comparison, we also assessed the sleep stage classification performance of classical CNNs. The corresponding results are discussed in detail below.

For the CNNs, the Normal CNN had the best classification accuracy and CK coefficient. However, when classification of each of the five sleep stages was compared using the sensitivity index, the performance of the Normal CNN was not ideal. For the waking stage, the VGG16 network had the best sensitivity with a value of 90.1%. For the N1 stage, VGG16 had the best sensitivity with a value of 28.4%. For the N2 stage, the Normal CNN had the best sensitivity with a value of 89.6%. For the N3 stage, VGG16 had the best sensitivity with a value of 80.9%. For the REM stage, AlexNet had the best sensitivity with a value of 85.7%. Additionally, the CK coefficient of VGG16 was only 0.0059 less than that of the Normal CNN. Thus, after this comprehensive consideration, these results suggest that the VGG16 network shows better performance than the Normal CNN. The Normal CNN had relatively high sensitivity only for the N2 stage, which might be due to the N2 stage class having the highest sample number. In the training phase, in order to minimize the cost function, this simple Normal CNN would tend to classify each sample into the class with the maximum number of samples (i.e., it suffered from an overfitting problem). Relying on its complex structure, the VGG16 network overcame the overfitting problem to some extent, and thus had a better classification performance for every sleep stage (quantified using the sensitivity index). Although GoogLeNet had the most complex structure, its sleep stage classification performance was worse. This might be due to the gradient attenuation problem, and also because of the performance saturation problem. In summary, when network structure became more complex, sleep stage classification performance of the CNNs increased and tended toward saturation.

For the LSTM networks, which considered the temporal information in each time-frequency spectral stack, LSTM 1 performed better than any of the CNNs. When more temporal information was considered, the performance of LSTM 2 and LSTM 3 increased, with the performance of LSTM 3 being better than all the networks mentioned above. Thus, similar to the CNNs, with more temporal information being taken into consideration, the sleep stage classification performance of the LSTM networks also increases and tended toward saturation. Compared with the CNNs, the LSTM networks not only had a simpler network structure, but also had the best sleep stage classification performance. Additionally, we also constructed a Normal CNN network with input mode 3 (i.e., the same as LSTM 3). Because the number of features that could be extracted increased, the CK coefficient of the Normal CNN network also increased. However, the classification performance of this network was only approximately the same as LSTM 1. This result further highlights the importance of time information between epochs for the final sleep stage classification performance.

Additionally, we also constructed LSTM networks with an input mode that considered the time information from the previous, current and future epochs (the initial input mode we used only considered the time information from the previous and current epochs). However, the performance of this input mode was approximately the same as the input mode 3 we used. Differences between these two input modes only occurred during epochs in which the sleep stage changed. However, for an entire night of sleep, this kind of epoch occurs very infrequently. This led to the training samples from these two input modes being almost the same, and thus, their performances were almost the same.

There might be two reasons that account for these results. The first is that manual sleep stage classification is based on certain specific wave, frequency or amplitude features. In contrast to other image classification problems, in which every image would be expected to have some features, single EEG data epochs may not have any features at some time points. For example, if an N2 stage feature occurred at time point *t*_1_, the technician would label the corresponding 30 s EEG data epoch as N2 sleep. Then, the technician would check the next 30 s epoch. If the next 30 s EEG data epoch did not have features from another sleep stage or did not have any features at all, the technician would go on checking the next 30 s EEG data epoch until other recognizable features of the stage occurred. Assuming that an N3 stage feature occurred at time point *t*_2_, then the technician would label the EEG data epoch between *t*_1_ and *t*_2_ as N2. Thus, some EEG data epochs which did not have any particular features would still be classified as being from a specific sleep stage. The second reason accounting for the results is that the CNNs only received the time-frequency spectral stack as an image and did not consider the temporal information within it. When temporal information was considered across the time-frequency spectral stack, the LSTM networks overcame this problem to some extent, and thus, had better performance than the CNNs. Considering these two reasons, the first might be said to be the more important. In addition, the rules for scoring sleep stages that were designed for a human eye to make sense of objectively measured analog signals may also account for these results. These rules were thoughtfully but arbitrarily defined, and are not always very specific, evidenced by the fact that two humans rarely agree 100% on any one record. Thus, the imperfect performance may also partially reflect this aspect of the scoring system itself.

There are still some limitations to this study. The first is that only a few datasets were used for testing; namely, only the SHHS2 dataset. Thus, future work will test our model on many more datasets. The second limitation is that we only used the time-frequency spectra as a classification feature. Thus, future work will use or combine other features to perform sleep stage classification.

In this study, an LSTM network with a time-frequency spectra as a feature was used to perform sleep stage classification. The results showed that an LSTM network with three consecutive spectral stacks as an input achieved the best performance. Compared with CNNs, LSTM networks can take temporal information into account, and thus, may be more suitable for sleep stage classification.

## Data Availability Statement

Publicly available datasets were analyzed in this study. This data can be found here: https://sleepdata.org/datasets/shhs.

## Ethics Statement

Ethical review and approval was not required for the study on human participants in accordance with the local legislation and institutional requirements. Written informed consent for participation was not required for this study in accordance with the national legislation and the institutional requirements.

## Author Contributions

ZX, JS, PL, XY, and WQ contributed to the conception and design. ZX contributed to the acquisition and analysis of data, and manuscript writing. ZX and XY contributed to the interpretation of the results.

## Conflict of Interest

The authors declare that the research was conducted in the absence of any commercial or financial relationships that could be construed as a potential conflict of interest.

## Footnotes

1 https://sleepdata.org/datasets/shhs
