# Supplementary material for: Sleep Stage Classification Using Time-Frequency Spectra From Consecutive Multi-Time Points
Source: Front Neurosci. 2020 Jan 28;14:14. doi: 10.3389/fnins.2020.00014 (PMC6997491; doi:10.3389/fnins.2020.00014)
Supplement: Supplementary file 1 [file Data_Sheet_1.PDF]

Supplementary Tables

Table S1. The structure for each CNN networks

	Normal CNN	AlexNet	VGG 16	GoogLeNet
**Stage 1**	conv3×3 32<br>conv3×3 32	conv11×11 96	conv3×3 64<br>conv3×3 64	conv7×7 64<br>(stride 2)
	conv3×3 32 (stride 2)	Max-pooling		
**Stage 2**	conv3×3 64<br>conv3×3 64	conv5×5 256	conv3×3 128<br>conv3×3 128	conv1×1 64<br>conv3×3 192
	conv3×3 64 (stride 2)	Max-pooling		
**Stage 3**	conv4×4 64	conv3×3 384<br>conv3×3 384<br>conv3×3 256	conv3×3 256<br>conv3×3 256<br>conv3×3 256	inception 3a 256<br>inception 3b 480
	—	Max-pooling		
**Stage 4**	—	—	conv3×3 512<br>conv3×3 512<br>conv3×3 512	inception 4a 512<br>inception 4b 512<br>inception 4c 512<br>inception 4d 528<br>inception 4e 832
	—	—	Max-pooling	
**Stage 5**	—	—	conv3×3 512<br>conv3×3 512<br>conv3×3 512	inception 5a 832<br>inception 5b 1024
	—	—	Max-pooling	Average-pooling
**Stage 6**	FC 5	FC 4096 (0.5)<br>FC 4096 (0.5)<br>FC 5	FC 4096 (0.5)<br>FC 4096 (0.5)<br>FC 5	dropout 0.5<br>FC 5

conv n×n m (stride y): Convolution layer, with kernel size as n×n, outputs number as m, and stride step as y×y. If it is not containing (stride y), then equals to (stride 1).

Max-pool: Max pooling layer, with kernel size as 3×3 and stride step as 2×2.

Average-pooling: Average pooling layer, with kernel size as 7×7 and stride step as 1×1.

FC m (n): Full connected layer, with output number as m and dropout rate as n.

inception IDX m: Inception layer, with index as IDX and output number as m.

Table S2. The structure of inception layer in GooLeNet

	A route	B route	C route	D route
**inputs**	inputs			
**route**	conv A 1×1	conv B1 1×1<br>conv B2 3×3	conv C1 1×1<br>conv C2 5×5	Max-pooling<br>conv D 1×1
**outputs**	concat outputs from all routes			

conv IDX n×n: Convolution layer, with index as IDX, kernel size as n×n and stride step as 1×1.

Max-pooling: Max pooling layer, with kernel size as 3×3 and stride step as 1×1.

concat: Concat layer.

Table S3. The output number of each inception layer in GoogLeNet

	conv A	conv B1	conv B2	conv C1	conv C2	conv D
**incept. 3a**	64	96	128	16	32	32
**incept. 3b**	128	128	192	32	96	64
**incept. 4a**	192	96	208	16	48	64
**incept. 4b**	160	112	224	24	64	64
**incept. 4c**	128	128	256	24	64	64
**incept. 4d**	112	144	288	32	64	64
**incept. 4e**	256	160	320	32	128	128
**incept. 5a**	256	160	320	32	128	128
**incept. 5b**	384	192	384	48	128	128

conv: Convolution layer; incept: Inception layer.
